# Conformal proctectomy with sphincter preservation retains acceptable defecation functions in very low rectal cancer male patients

**DOI:** 10.3389/fonc.2024.1478467

**Published:** 2024-11-07

**Authors:** Weijie Chen, Xiao Zhang, Xiaoyuan Qiu, Jiaolin Zhou, Guole Lin

**Affiliations:** Department of General Surgery, Peking Union Medical College Hospital, Chinese Academy of Medical Sciences, Beijing, China

**Keywords:** rectal cancer, total mesorectal excision, anal function, clinical trial, quality of life

## Abstract

**Background:**

Conformal proctectomy with sphincter preservation (CPSP) is designed to preserve the rectal wall as much as possible in very low rectal cancer patients. Evaluations of anal function and quality of life outcomes are lacking.

**Methods:**

This study included male patients with very low (≤ 5 cm from the anal verge) rectal adenocarcinoma between January 1, 2020, and January 1, 2022. A LARS score questionnaire survey and EORTC-QLQ-CR38 questionnaire survey were administered.

**Results:**

A total of 21 very low rectal cancer patients were enrolled in follow-up. The average age of the patients was 56.7 years, the tumors were 1.9 ± 0.6 cm in size, and the distance from the anal verge was 4.8 ± 0.5 cm. All patients were followed up, and the mean follow-up period was 2.7 ± 0.5 years. The LARS score increased significantly from 4.1 ± 2.8 before surgery to 19.1 ± 6.0 at the 1^st^ year after surgery (*P* < 0.001) and then decreased to 13.1 ± 4.2 (*P* < 0.001) at the 2^nd^ year. The quality of life of patients was also lower at the 1^st^ year after surgery (61.1 ± 9.6 vs. 74.2 ± 11.2, *P* < 0.001) and was restored at the 2^nd^ year after surgery (80.6 ± 11.9 vs. 74.2 ± 11.2, *P* = 0.029). During standard follow-up at the outpatient department, no rectal tumor relapse was confirmed in these patients, although 2 patients were found to have suspected recurrence of local lymph node metastasis.

**Conclusions:**

These results suggest that the CPSP technique preserves acceptable defecation function and is a safe and feasible option for male patients with very low rectal cancer.

**Clinical trial registration:**

https://www.chictr.org.cn/, identifier ChiCTR2100052094.

## Introduction

Rectal cancer is a common malignant tumor with a high mortality rate. A tumor located within 5 cm from the anal verge is classified as very low rectal cancer. Traditionally, very low rectal cancer was treated by abdominoperineal resection (APR) without preserving the anus. With the advancements in surgical oncology and instrumentation, intersphincteric resection (ISR) and coloanal anastomosis (CAA) have been developed to remove the very low rectal cancer and preserve the anus. The incidence of recurrence and long-term survival of patients seem not to be impacted by the reduction in the distal resection margin (1). However, the poor anal function was found during the follow-up period in patients who underwent ISR and most functional problems were due to the removal of the internal anal sphincter and destruction of the autonomic nerves. Poor functional problems lead to a significant decrease in postoperative quality of life (QoL).

Conformal proctectomy with sphincter preservation (CPSP) was recently introduced to clinical surgical practice (2). The conformal incision line is designed according to the tumor’s location and size to preserve as much of the distal rectum, dentate line and internal anal sphincter on the side opposite to the tumor as possible. The internal anal sphincter and the dentate line are important parts of the anal sphincter complex that preserve anal functions. Unlike transanal total mesorectal excision, CPSP does not involve pulling the rectum out of the anus through the rectal lumen. This technique preserves more of the dentate line and distal opposite rectal wall without enfolding the rectal wall, and the intersphincteric space remains undisturbed, which prevents injuries to the numerous nerve fibers it contains (2).

Moreover, male patients have a narrow pelvis and a tight anus. It is not easy to dilate the anus to perform transanal total mesorectal excision or to pull out the rectum through anus. An increasing number of male patients choose CPSP as their treatment. The aim of the present study was to follow low rectal cancer male patients after CPSP to evaluate the effectiveness of preserving anal function and quality of life.

## Methods

### Study design and participants

**Figure 1:**
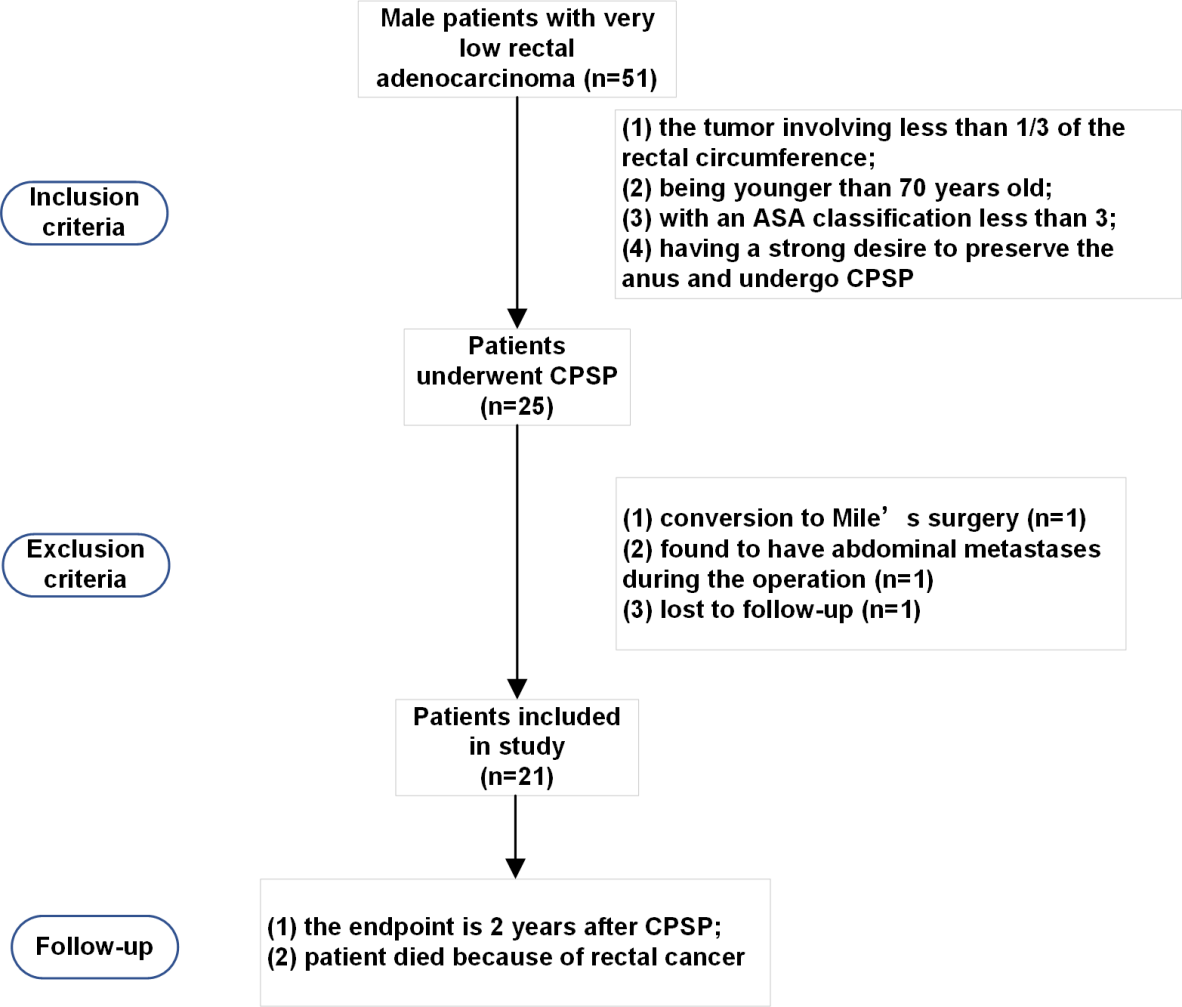
Patient selection for conformal proctectomy with sphincter preservation.

Between January 1, 2020, and January 1, 2022, 51 male patients with very low (≤ 5 cm from the anal verge) rectal adenocarcinoma were referred for operative treatment. Based on our previous experience and published literature, patients who met the following inclusion criteria were considered for laparoscopic CPSP: (1) the tumor involving was less than 1/3 of the rectal circumference; (2) patient was younger than 70 years old; (3) the American Society of Anesthesiologists (ASA) classification of patient was less than 3; (4) patient had a strong desire to preserve the anus and undergo CPSP. The exclusion criteria were as follows: (1) conversion to Miles’ surgery; (2) R2 resection; (3) total or partial pelvic exenteration; and (4) the use of drugs that could affect defecation function. A total of 25 patients were found to be suitable for CPSP and are the basis of this study (**Figure 1**). All patients provided informed consent. Demographic information, comorbidities, and the medical history of the participants were collected. The study was registered in the Chinese Clinical Trial Registry under the registration number ChiCTR2100052094 and approved by the Ethics Committee of Peking Union Medical College Hospital (Ethics review approval No. JS-3361).

### CPSP procedures

**Figure 2:**
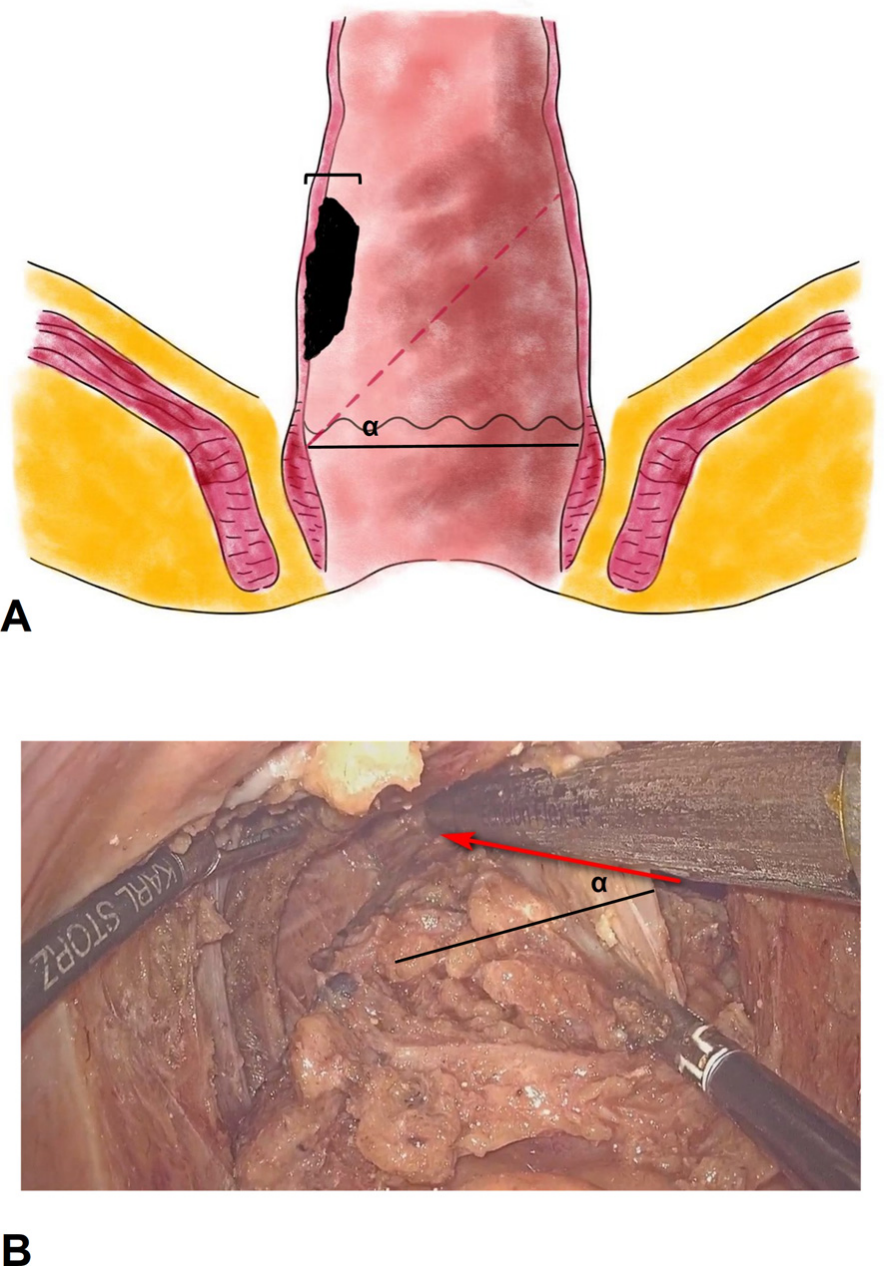
The sketch and operative picture of conformal proctectomy with sphincter preservation. **(A)**, the sketch of conformal proctectomy with sphincter preservation. α, the angle between the cutting line and the dentate line. We recommend that the angle is 45°, and less than 60°. The width tumor involved should be less than 1/3 of rectal circumference. **(B)**, the operative picture of conformal proctectomy with sphincter preservation. The rectum was mobilized according to the principle of total mesorectal excision until it reached the entrance of the intersphincteric space. Pull and stretch the rectum using laparoscopic bipolar forceps, and place endoscopic stapler at an angle (α) in the pelvic cavity to preserve more of the rectal wall.

The sigmoid colon was initially mobilized, the inferior mesenteric artery was ligated at its origin, and the autonomic nerves were carefully preserved. The rectum was mobilized according to the principle of total mesorectal excision (TME) until it reached the entrance of the intersphincteric space. The conformal distal incision line was designed according to the location and size of the tumor. The aim was to preserve as much of the lower rectum, dentate line and internal anal sphincter on the side opposite the tumor as possible (**Figure 2**). The angle between the cutting line and the dentate line was approximately 45°. The distal dissection line was made at least 1 cm below the inferior tumor margin under direct vision. The intersphincteric space was left undisturbed as much as possible to prevent injury to the numerous nerve fibers contained therein. End-to-end anastomosis was performed with a 25 mm circular stapler (CDH25, Johnson & Johnson, USA) under laparoscopy. The stapler was inserted as high up in the rectal stump as possible and tiled to the tumor side. The aim was to make the anastomosis on the rectal wall on the opposite side and to try to keep the dentate line and internal anal sphincter unbroken.

Temporary ileostomy was routinely performed and restored approximately 6 months later if there was no significant anastomotic stricture or anal dysfunction. The anastomotic status could be assessed by endoscopy or gastrointestinal contrast examination. Inject the vascular contrast agent (Omnipaque) mixed with 100ml of saline through a silicone tube (or catheter) into the colon 20cm above the colorectal (or coloanal) anastomosis, and then a standing X-ray examination or CT scan was performed. Anal function can also be evaluated by injecting 100ml saline into the colon above anastomosis. The patient’s health status is fully recovered to baseline, and inflammation has subsided with softening of adhesions a few months after surgery. Then the ileostomy was restored.

The following surgery-related information was collected: blood loss, operation duration, postoperative hospital stay duration, tumor size, distance from the anal verge, distal resection margin length, pathological T stage, pathological N stage, and tumor differentiation status. Patients with pathological stage III or stage II tumors with high-risk features received the postoperative CapeOx or mFOLFOX6 regimen as adjuvant chemotherapy. Postoperative radiotherapy was performed in patients who had not received preoperative radiotherapy according to the following criteria: (1) pathological result ≥ N1b or circumferential resection margin (CRM) positive; (2) T3 or T4 disease; and (3) distal margin too short (usually less than 0.3 cm).

### Defecation function assessment

Bowel dysfunction of patients was assessed via the low anterior resection syndrome (LARS) score questionnaire. LARS is defined by at least one of the following symptoms resulting in at least one of the following consequences that occur after a sphincter-sparing resection of the rectum (3). The symptoms include unpredictable bowel function, altered stool consistency, increased stool frequency, repeated painful stools, emptying difficulties, urgency, incontinence and soiling. The LARS questionnaire consists of 5 items: incontinence for flatus and liquid stools, defecation frequency, stool clustering, and urgency (4). Each item was graded from 3 to 16 points. The total score ranges from 0 to 42, and a lower score indicates better defecation function: no LARS (0-20), minor LARS (21-29), and major LARS (30-42).

### Life quality assessment

The EORTC-QLQ-CR38 questionnaire survey was also conducted before and after surgery. The EORTC-QLQ-C30 questionnaire is specific for colorectal cancer patients (5). The EORTC QLQ-C30 questionnaire includes 15 items: physical function (5 questions), role function (2 questions), emotional function (4 questions), cognitive function (2 questions), social function (2 questions), global health (2 questions), fatigue (3 questions), nausea and vomiting (2 questions), pain (2 questions), dyspnea (2 questions), insomnia (1 question), appetite loss (1 question), constipation (1 question), diarrhea (1 question), and financial difficulties (1 question). Each question was graded from 0 to 4 points. The raw score was then standardized to range from 0 to 100. A lower score in the functional domain indicates worse function, and a lower score in the symptom domain indicates better symptoms.

### Follow-up

Each patient underwent standard follow-up at the outpatient department from the first month after surgery. Tumor relapse was screened for with serum tumor markers and CT examination of the chest, abdomen, and pelvis every 6 months during the first 2 years. Moreover, assessments of defecation function and QoL were conducted at the outpatient department or via telephone.

The endpoint of the study is 2 years after CPSP. The second endpoint is that the patient died because of rectal cancer.

### Statistical analysis

Continuous variables are presented as the means ± standard deviations and were analyzed with paired t tests to compare the values before and after surgery. Categorical variables are presented as numbers (percentages) and were analyzed with the chi-square test or Fisher’s exact test. A two-sided *P* value of less than 0.05 was considered statistically significant. Statistical analyses were performed with SPSS for Windows, version 20.0 (SPSS, Chicago, IL, USA). The sample size was calculated using G Power 3.1.9.7 (Universität Düsseldorf), with the α err prob of 0.05 and the power (1-β err prob) of 0.95, then the calculated sample size was 15.

## Results

### Demographic characteristics of the included patients

Between January 1, 2020, and January 1, 2022, a total of 25 very low rectal cancer patients were enrolled in the study. Two patients were excluded because they were found to have abdominal metastases during the operation, 1 patient underwent conversion to Mile’s surgery, and 1 patient was lost to follow-up. Ultimately, 21 patients who underwent CPSP were followed up. The demographic characteristics of the patients are summarized in **Table 1**.

**Table 1:** The clinical characteristics of patients.

Characteristics	CPSP Patients
n	21
Age (years)	56.7 ± 9.4
BMI (kg/m^2^)	24.5 ± 3.1
Previous history	
Diabetes	4 (19.0%)
Coronary heart disease	3 (14.3)
Abdominal operation	2 (9.5%)
Tumor characteristic	
Tumor size (cm)	1.9 ± 0.6
T category	
T1	2 (9.5%)
T2	5 (23.8%)
T3	13 (61.9%)
T4	1 (4.8%)
N category	
N0	7 (33.3%)
N+	14 (66.7%)
Distance from the anal verge	4.8 ± 0.5
Anterior wall	6 (28.6%)
Lateral and posterior wall	15 (71.4%)
Preoperative neoadjuvant therapy	15 (71.4%)
Xelox	15 (71.4%)
Radiation (45Gy)	14 (66.7%)

The average age of the patients was 56.7 years, and their body mass index was 24.5 ± 3.1 kg/m^2^. The tumors were 1.9 ± 0.6 cm in size; 85.7% of them were classified as T2 or T3, 71.4% of them were distributed on the lateral or posterior wall, and the distance between the tumor distal margin of the tumor and the anal verge was 4.8 ± 0.5 cm. A total of 66.7% of the patients had positive local lymph node metastasis on imaging. A total of 71.4% of the patients accepted the chemotherapy regimen with Xelox, and 66.7% of the patients accepted treatment with 45 Gy radiation.

### Perioperative outcomes

The operative duration was 121.7 ± 28.3 minutes, and the blood loss volume was 43.3 ± 27.4 ml. Although preventive stoma formation was performed, 1 patient (4.8%) experienced anastomotic leakage. One patient (4.8%) experienced urine retention. According to the Clavien-Dindo classification, 2 patients (9.5%) were classified as having Grade II complications. The postoperative hospital stay duration was 7.1 ± 1.8 days (**Table 2**).

**Table 2:** Perioperative outcomes and pathological characteristics.

Characteristics	CPSP Patients (n=21)
Duration of operation (minutes)	121.7 ± 28.3
Blood loss (ml)	43.3 ± 27.4
Complications	2 (9.5%)
Anastomotic leakage	1 (4.8%)
Stoma-related complications	0
Urine retention	1 (4.8%)
Ileus	0
Clavien-Dindo grade	
I-II	2 (9.5%)
III-IV	0
Postoperative hospital stay (days)	7.1 ± 1.8

### Pathological assessment

The margin clearance of the resected specimen was given special attention. The circumferential resection margin was negative, and the mesorectum was complete. The distance between the incisal margin and distal tumor margin was 1.1 ± 0.6 cm. The postoperative pathological results revealed that the main proportion of tumors were moderately differentiated (57.1%) (**Table 3**). A total of 61.9% of the tumors were classified as T1 or T2, and 85.7% of the patients were found to have local lymph nodes that were negative for metastasis.

**Table 3:** Pathological properties.

Characteristics	CPSP Patients (n=21)
Circumferential resection margin positive (n)	0
Completeness of mesorectum (n)	21 (100%)
Distal resection margin length (cm)	1.1 ± 0.6
Tumor differentiation (n)	
Well	5 (23.8%)
Moderate	12 (57.1%)
Poor	4 (19.0%)
Pathological T category (n)	
T1-2	13 (61.9%)
T3-4	8 (38.1%)
Pathological N category (n)	
N0	18 (85.7%)
N+	3 (14.3%)

### Quality of life

**Figure 3:**
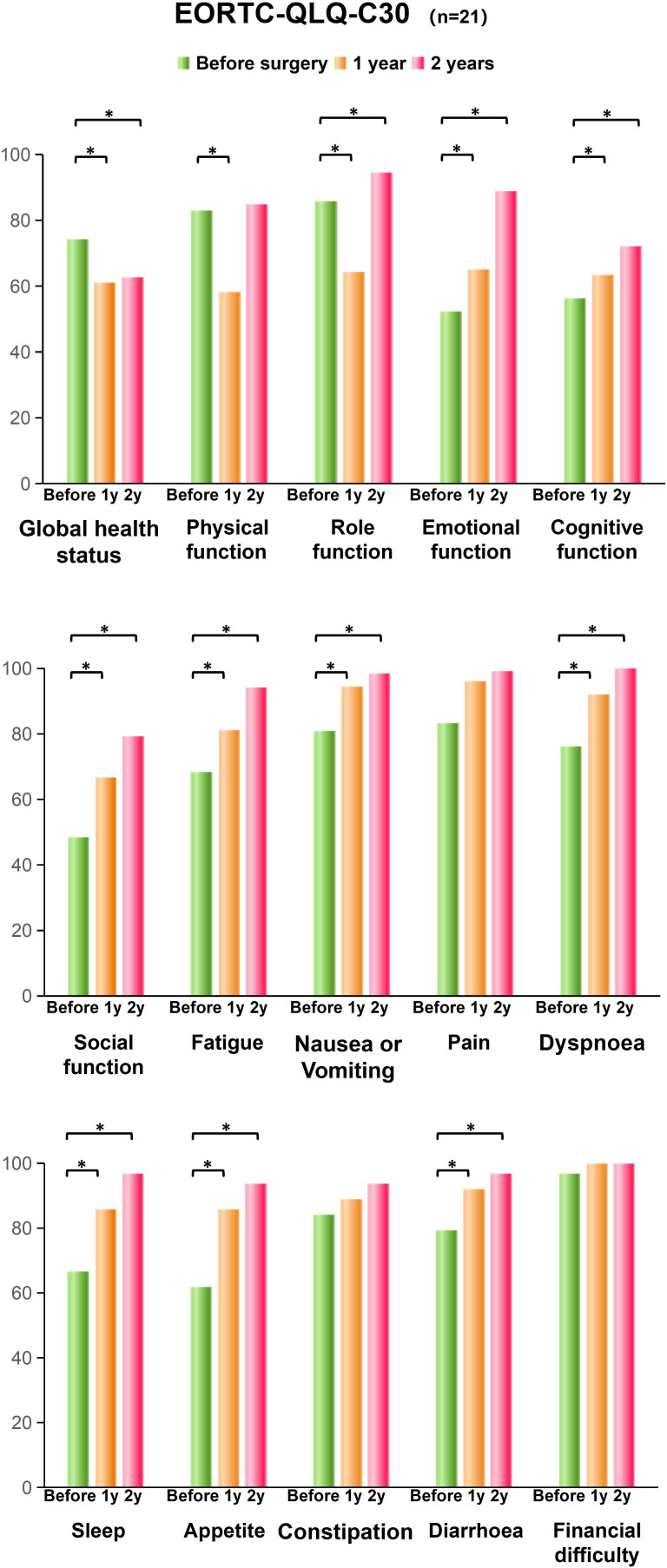
EORTC-QLQ-C30 scores of the conformal sphincter preservation operation patients. The global health status, functional scales, symptom scales and single item measures before and after surgery were shown. An asterisk indicates significant differences. * means significant difference, *P* < 0.05.

Of the 22 patients who were followed up, 21 patients (95.5%) responded to our interviews. All patients were followed up for more than 2 years. The follow-up period was 2.7 ± 0.5 years. Among them, 7 patients were followed up for more than 3 years. The LARS score significantly increased from 4.1 ± 2.8 before surgery to 19.1 ± 6.0 at the 1^st^ year after surgery (*P* < 0.001), and then reduced to 13.1 ± 4.2 at the 2^nd^ year after surgery (*P* < 0.001, **Table 4**). The quality of life score of patients also decreased from 74.2 ± 11.2 before surgery to 61.1 ± 9.6 at the 1^st^ year after surgery (*P* < 0.001) and then rose to 80.6 ± 11.9 at the 2^nd^ year after surgery (*P* = 0.029, **Figure 3**). The subscales physical functioning (*P* < 0.001), role functioning (*P* < 0.001), emotional functioning (*P* < 0.001), cognitive functioning (*P* < 0.001), social functioning (*P* < 0.001), fatigue (*P* < 0.001), nausea/vomit (*P* < 0.001), dyspnea (*P* < 0.001), sleep (*P* < 0.001), appetite (*P* < 0.001) and diarrhea (*P* < 0.001) subscale scores also significantly differed during the 2-year follow-up period. However, the constipation (*P* = 0.31), pain (*P* = 0.18) and financial difficulties (*P* = 0.14) subscale scores were not significantly different before surgery and after surgery.

**Table 4:** The long-term functional outcomes.

Characteristics	CPSP Patients (n=21)
Defecation functions (LARS)	
Before surgery	4.1 ± 2.8
1 year after surgery	19.1 ± 6.0
2 years after surgery	13.1 ± 4.2
Life quality assessment (EORTC CR38)	
Before surgery	74.2 ± 11.2
1 year after surgery	61.1 ± 9.6
2 years after surgery	80.6 ± 11.9

Data are mean ± SD. low anterior resection syndrome (LARS) score.

Moreover, at the 2^nd^ year after surgery, physical function was not significantly different from that before surgery (*P* = 0.51). The subscales scores for role functioning, emotional functioning (*P* < 0.001), cognitive functioning (*P* < 0.001), social functioning (*P* < 0.001), fatigue (*P* < 0.001), nausea/vomit (*P* < 0.001), dyspnea (*P* < 0.001), sleep (*P* < 0.001), appetite (*P* < 0.001) and diarrhea (*P* < 0.001) improved 2 years after surgery.

### Oncological outcomes

During the 2-years follow-up period at the outpatient department, no rectal tumor relapse was found in these patients, although 2 patients (9.5%) experienced suspected local lymph node metastasis recurrence.

## Discussion

Anal function is considered a quality marker for low rectal cancer surgery. Patients with tumors close to the anal verge have a greater risk of defecation dysfunction after laparoscopic anterior resection (4, 6). Common symptoms are frequent bowel movements, fecal incontinence, urgency and clustering of stools. Patients who undergo ISR generally have worse incontinence, and a greater proportion of ISR patients develop low anterior resection syndrome (7, 8). Fecal urgency is observed in up to 58.8% of patients (9), and the mean number of bowel movements in a 24-hour period was 2.7 (10). At 10 years after ISR, approximately 18% of patients still require a stoma (11).

Colorectal or coloanal anastomosis must be reestablished if the anus needs to be retained. Coloanal anastomosis often leads to frequent or fragmented stool patterns for at least one year postoperatively, as the internal anal sphincter complex is essential for anal function (12). The internal anal sphincter is a thickened, circular smooth muscle layer innervated by the enteric nervous system. It is tonically contracted, which accounts for 80% to 85% of the anal canal resting pressure, helping to maintain continence (13). Reduced internal anal sphincter resting pressure may result in incontinence (14).

Colorectal anastomosis is only successful if there is sufficient rectal reserve. CPSP is performed to attempt to retain the rectal wall against the tumor and achieve an oblique colorectal anastomosis using the rectal wall against the tumor. The ultimate goal is to protect the internal anal sphincter complex by creating the fashioned anastomosis. Usually, the anastomosis ring can be located 1-2 cm above the dentate line. And the lower rectum does not need to be inverted and pulled out through anus during CPSP (15). *In situ* excision can preserve more of the rectal wall. Although G.Sun et al. Described a similar concept for conformal resection of the rectal wall (16), they did not mention the detailed procedure or particular factors that need careful attention. The “Z”-shaped resection line shown in their schematic diagram necessitates the use of more than 3 staplers and is hard to perform during surgery. The width of rectal wall is 3 cm approximately, and the smallest stapler size is 2 cm in our country. At the location less than 5 cm from the anal verge, it is hard to place the stapler perpendicular to the rectum in the male narrow pelvic cavity (17). By contrast, the inclined cutting line is relatively easy to perform, and fewer stapler is used. The key factor affecting CPSP is the size of the tumor, which needs to be less than 1/3 of the rectal circumference. Then, the angle between the cutting line and the dentate line can be less than 60°. We recommend that the angle be 45°. If the angle is more than 60°, the resident rectal wall might lack a blood supply, resulting in anastomosis leakage. If the angle is less than 30°, the rectal wall against the tumor is less well preserved, and anal function might be affected. Transanal Transection and Single-Stapled Anastomosis (TTSS) is also a promising surgical technique for very low rectal cancer. It could preserve at less 0.5cm rectal wall by Single-stapled anastomosis and avoid a double-stapled anastomosis (18). However, TTSS requires surgical expertise and careful patient selection to ensure its success. A high level of skill to perform the transanal transection accurately and to create a secure anastomosis is needed.

In the present study, we found that patients who underwent CPSP had better global quality of life after surgery. Our findings regarding symptom scales scores also revealed better anal function without constipation or diarrhea. Several studies have shown the deterioration of bowel function in patients treated with surgery plus radiotherapy compared with that in patients treated with surgery alone (19). Notably, despite a significantly larger proportion of CPSP patients receiving neoadjuvant radiotherapy, this did not affect anal function in these patients. Moreover, CPSP yields good oncological outcomes. During the more than 2-year follow-up period, 2 patients were found to have suspected local lymph node metastasis relapse, which is in accordance with a previous report (20). In terms of tumor biology, a 1 cm negative distal margin in conjunction with the TME may be acceptable (21), particularly in the context of neoadjuvant chemoradiation therapy (22).

Our study design included patients at a minimum of 2 years postsurgery, which allowed us to ignore temporary disturbances or stoma function in the early postoperative phase. To verify this, we investigated the association between anal function and time since surgery. Bowel function at 2 years after surgery was better than that at 1 year after surgery (*P* = 0.005). With the complement of the internal anal sphincter, anal function could be restored to some extent after ostomy restoration. Similarly, after ileostomy was restored, the QoL of patients improved significantly, especially their emotional functioning, role functioning and social functioning.

A weakness of our study is its cross-sectional design and limited size, which might preclude any conclusions regarding causality. However, we described a practical procedure and demonstrated that it can effectively resolve very low rectal cancer without necessitating the removal of the anus. Furthermore, the CPSP technique preserves acceptable defecation function and QoL, which is particularly important given the increasing focus on functional recovery and quality of life in rectal cancer patients. On the basis of our findings, we suggest that CPSP is a safe and feasible option for male patients with very low rectal cancer.

## Data Availability

The original contributions presented in the study are included in the article/supplementary material. Further inquiries can be directed to the corresponding author.
